# Effects of Milk Thistle Extract Supplementation on Performance, Egg Quality, and Liver Pathology of Laying Hens’ Fed Diets Lacking Supplemental Choline Chloride

**DOI:** 10.3390/vetsci12020077

**Published:** 2025-01-21

**Authors:** Fotis Gousias, Ioanna Stylianaki, Ilias Giannenas, Theodoros Kallitsis, Nikolaos Papaioannou, Efstratios Chaitidis, Clare Squires, Georgios Arsenos, Vasilios Tsiouris, Georgios A. Papadopoulos

**Affiliations:** 1 Laboratory of Animal Husbandry, Faculty of Veterinary Medicine, Aristotle University of Thessaloniki, 541 24 Thessaloniki, Greece; tgkallitsis@vet.auth.gr (T.K.); squires@vet.auth.gr (C.S.); arsenosg@vet.auth.gr (G.A.); geopaps@vet.auth.gr (G.A.P.); 2 Laboratory of Pathology, Faculty of Veterinary Medicine, Aristotle University of Thessaloniki, 541 24 Thessaloniki, Greece; stylioan@vet.auth.gr (I.S.); nikpap@vet.auth.gr (N.P.); echaiti@vet.auth.gr (E.C.); 3 Laboratory of Nutrition, Faculty of Veterinary Medicine, Aristotle University of Thessaloniki, 541 24 Thessaloniki, Greece; igiannenas@vet.auth.gr; 4 Unit of Avian Medicine, Clinic of Farm Animals, Faculty of Veterinary Medicine, Aristotle University of Thessaloniki, 546 27 Thessaloniki, Greece; biltsiou@vet.auth.gr

**Keywords:** laying hens, milk thistle, egg quality, liver health

## Abstract

The current study evaluated the effects of milk thistle extract supplementation in laying hens’ fed diets lacking choline chloride addition. A total of 60 Isa-brown laying hens were randomly allocated into T1: control diet, 0% extract supplementation; T2: control diet with 1% extract; T3: control diet with 2.5% extract; and T4: control diet with 4% extract. Egg quality parameters, yolk lipid oxidation, malondialdehyde (MDA) content, and fatty acid profile were assessed. Livers were examined grossly and histologically to evaluate hepatocellular lesions such as vacuolization (lipidosis), reticular stromal architecture, the amount of collagenous connective tissue, and vascular wall changes. Groups T3 and T4 showed darker yolks compared to both control group and T2 (*p* = 0.001) and redness of the egg yolk (*p* < 0.001). The MDA was lowest in T2 group which had improved gross appearance with lower degrees of hepatic vacuolization than other groups. Liver discoloration was milder in T3 (43.8%) compared to the T1 and T4 groups (18.8% and 12.5%, respectively, *p* = 0.013). Reticulin loss was correlated with the degree of hepatic vacuolization (r = 0.751, *p* < 0.001). Supplementation with MT extract in diets lacking choline chloride may influence certain egg quality indices and liver gross macroscopic lesions in laying hens.

## Simple Summary

This study investigated the effects of milk thistle (*Silybum marianum*) extract supplementation on egg quality, yolk lipid oxidation, and liver health in laying hens’ fed diets lacking supplemental choline chloride. Fatty liver hemorrhagic syndrome (FLHS) is a metabolic disorder common in caged hens, exacerbated by high-energy diets and choline deficiency, which impairs lipid metabolism in the liver. The results showed that yolk coloration may be increased in the case of higher levels of supplementation, while supplementation of 1% extract had lower malondialdehyde (MDA) levels in yolks. Additionally, liver histopathology revealed milder vacuolization in the 1% and 2.5% supplementation groups compared to the control. The study concluded that milk thistle extract, particularly at 1% supplementation, may contribute to liver health and reduce oxidative stress in laying hens’ fed diets lacking choline chloride supplementation. These findings are valuable as they suggest a potential natural supplement to improve liver function and egg quality in poultry production, contributing to animal welfare and more sustainable farming practices.

## 1. Introduction

Global egg production has shown a remarkable increase over approximately the last 30 years [1]. Although alternative egg production systems have emerged recently in response to consumers’ concerns about hen welfare, the great majority comes from caged hens [2]. Fat liver hemorrhagic syndrome (FLHS) is a prevalent metabolic disorder that is the primary cause of mortality in commercially raised caged layers, particularly in conditions with high production rates [3,4]. Excessive feed intake, high in energy with an unbalanced amino acid sequence, has been speculated as a potential factor that can lead to fat storage in the liver, causing fatal hemorrhages through its oxidation [4]. Fatty liver hemorrhagic syndrome (FLHS) leads to the abrupt death of birds due to liver rupture and hemorrhaging [5,6]. The hepatic tissue of the hen is crucial in the process of synthesizing and metabolizing lipids. Fat synthesis in birds is significantly higher in the hepatic tissue compared to the adipose tissue [7,8]. Consequently, investigating strategies to prevent, mitigate, and manage FLHS in laying hens has emerged as a crucial area of research in the poultry sector. In this context, dietary supplements with antioxidant and anti-inflammatory properties have been proposed as potential preventative measures.

Milk thistle (MT), widely known as *Silybum marianum*, has been the most extensively studied plant in the treatment of liver diseases [9]. The therapeutic characteristics of MT are mainly attributed to the mixture of flavonolignans, known as silymarin [10]. Silymarin consists predominantly of silybin, the main biologically active component that accounts for almost 60% of its composition, and secondarily of silychristin (20%), silydianin (20%), and isososylibin (5%) [11,12]. Research has demonstrated that silymarin exhibits antioxidant, anti-inflammatory, and anti-fibrotic properties that aid in the repair of injured liver cells [13,14]. The primary mechanisms of silymarin’s antioxidant effect have been identified as the suppression of lipid peroxidation of cell membranes and reduced hepatic tissue damage caused by lipid peroxides [15,16]. It was demonstrated that, in contrast to other oxidants such as hydrogen peroxide, superoxide anion radical, and hypochlorous acid, silymarin interacted quickly with hydroxyl radical [14]. Silymarin may inhibit the nuclear transcription factor NF-κB, which is activated by pro-inflammatory cytokines such as IL-1b and TNF-α, contributing to its anti-inflammatory impact [17]. The inhibition of hepatic stellate cell (HSC) conversion into myofibroblasts, which is thought to be a key component of fibrosis, has been linked to silymarin’s anti-fibrotic action [18]. Silymarin has the potential to heal injured hepatocytes by enhancing protein synthesis in the hepatic tissue, although the exact mechanism is not yet fully understood [12]. Elsewhere, silybin appeared to boost the production rate of ribosomes, hence enhancing protein synthesis [19].

Choline chloride dietary supplementation has been widely used as the primary hepatoprotective dietary ingredient, given its established favorable effect in reducing hepatic lipid accumulation and the presence of fatty liver in laying hens [20,21,22]. The use of choline supplementation, such as choline chloride, during the laying period significantly improved egg production and egg size [23,24]. Moreover, choline deficiency in humans led to human steatosis, increasing predisposition to liver damage that was ameliorated by the dietary choline restoration [25,26]. To date, MT dietary supplements efficacy has only been investigated in laying hen diets that were supplemented with choline chloride, but not in diets lacking choline chloride supplements. Specifically, Kavan et al. (2023) [27] found enhanced egg weight and egg production by supplementing 200 mg/kg of MT oil. On the other hand, Karamali et al. (2020) [28] found no effect of 200 mg/kg MT powder administration on egg production, but reported a better feed conversion ratio (FCR). In addition, Quarantelli et al. (2009) [29] showed an improved egg-laying rate and feed conversion ratio together with changes in the egg lipid content in laying hens in the early productive stage. Elsewhere, dietary supplementation in powder form did not influence yolk lipid peroxidation levels [30].

Yet, it is evident that the effects of MT extract with silymarin as the main active ingredient have not been investigated in laying hens’ diets lacking choline chloride supplementation. Based on the properties of silymarin, it can be hypothesized that it could counteract lipid accumulation and associated disorders in the liver, compensating for cases of diets lacking choline chloride supplementation. It was anticipated that the margin of the effect of silymarin supplementation would be more evident during the post-peak laying hen period. Therefore, in this study, the effects of MT extract administration on laying hen production performance and egg quality were evaluated, with a particular emphasis on liver gross pathology, histopathology, and immunohistochemistry markers in the context of diets lacking choline chloride supplementation.

## 2. Materials and Methods

### 2.1. Experimental Design and Dietary Treatments

The study occurred in an experimental facility in the American Farm School of Thessaloniki, Greece. In total, 60 Isa brown laying hens at a post-peak period (55 weeks old) were randomly allocated in 4 treatments and were fed on the following diets for 8 weeks: T1 (0%), T2 (1%), T3 (2.5%) and T4 (4%). The birds were individually installed in 40 cm × 40 cm cages, which provided more space per hen (1600 cm^2^), compared to the minimum requirements of a recent EU directive (at least 750 cm^2^ of cage area per hen) [31] (Directive 1999). Each treatment consisted of 15 replicate cages and the diets were formulated based on the Isa brown commercial guide. The main ingredients and calculated nutrient analysis of the diets are shown in Table 1. All diets were not supplemented with choline chloride. The total choline content of diets provided by the feed ingredients used was also calculated. There was a 2-week adaptation period before the onset of the experimental period in which the layers were initially fed on the basal diet during the first week and on a 50:50 ratio of the basal and each experimental diet during the second week. The layers were offered ad libitum access to water and feed, since each cage possessed its own water dispenser and a feeder pan placed in the front side of each one. Their floors were inclined to allow the daily collection and record of the egg production. The ambient temperature was around 20 °C, the relative humidity was between 50 and 70%, and the light duration was 14 h per day. The experimental period lasted 8 weeks and the egg samples were collected at the beginning, the middle, and the end of this period. These samples were analyzed immediately for egg quality parameters and the yolks were stored in individual containers at −20 °C for further analysis.

### 2.2. Performance of Laying Hens

The total feed consumption was measured weekly, and the quantity of laid eggs and their weight were measured daily. Thus, the egg production rate was calculated, along with the daily egg mass as the product of mean egg weight times, the production rate, and the average daily feed intake per hen. Furthermore, the feed conversion ratio per egg was calculated as the ratio between the average feed intake per hen and the egg mass.

### 2.3. Egg Quality Parameters

In total, 144 eggs were collected for the evaluation of the quality parameters. This accounts for 12 eggs per group (48 in total) on weeks 1, 4, and 8, respectively. The quality parameters are as follows: egg weight, yolk weight, albumen weight, eggshell weight, eggshell thickness, longitudinal and transverse axes, shape index, eggshell color, yolk color, Haugh units. The DSM YolkFanTM scale was used for the Yolk color score and the Chroma meter CR-410 (Konica Minolta, Osaka, Japan) using the L*a*b* color values was utilized for the instrumental assessment. The L* value illustrates lightness (0 = black, 100 = white), the a* value represents redness (−100 = green, 100 = red) and the b* value detects yellowness (−100 = blue, 100 = yellow). Overall period values were calculated, taking into account the measurements from all experimental weeks.

### 2.4. Yolk Lipid Oxidation

In total, 8 eggs were used per treatment at the end of the 1st, 4th and 8th week for the evaluation of the yolk lipid oxidation, which was assessed by TBARS assay (Thiobarbituric Acid Reactive Substances). In brief, 1 g of yolk was added to in a conical centrifuge tube with 8 mL of 5% Trichloroacetic acid (TCA) aqueous solution and 5 mL of 0.8% Butylated hydroxytoluene (BHT) solution in hexane and then homogenized using an Ultra-Turrax device (model T25-S5, IKA-Labortechnik, Janke & Kunkel, GMBH, Stuttgart, Germany) and a Vortex apparatus (REAX 1R model, Heidolph, Schwabach, Germany). The tubes were then centrifuged at 3000 rpm for 3 min. Afterwards, 2.5 mL of the bottom layer was collected and transferred into a new tube with the addition of 1.5 mL of 0.8% Thiobarbituric Acid aqueous solution. The tubes were placed in a water bath for 30 min at 70 °C and then they were cooled down with tap water. Finally, the absorbance was measured in a spectrophotometer at 532 nm and the results were measured as ng of Malondialdehyde (MDA) per gram of yolk (ng MDA/g yolk).

### 2.5. Yolk Total Phenolic Content (TPC)

Ιn total, 12 eggs per treatment at the end of the 8th week were used for the evaluation of total phenolic content, which was measured by the Folin–Ciocalteu assay, according to the protocol described by Shang et al. (2020) [32].

### 2.6. Egg Yolk Fatty Acid Profile

The fatty acid profile in egg yolk was analyzed using gas chromatography. Specifically, 10 μL of 200 mg/mL pentadecanoic acid (Sigma, St. Louis, MO, USA) in chloroform as internal standard were mixed thoroughly with approximately 50 mg of egg yolk (weighed to the first decimal point of the milligram) in a test tube before extracting lipids according to Folch et al. [33].

### 2.7. Gross Evaluation, Histopathology, and Immunohistochemistry

At the end of the experimental period hens (*n* = 7 per treatment) were humanely euthanized by using CO_2_ at the premises of the Unit of Avian Medicine (Faculty of Veterinary Medicine, Aristotle University of Thessaloniki, 546 27 Thessaloniki, Greece). All livers collected from laying hens (*n* = 7 per treatment) underwent a standardized processing protocol, including gross evaluation, comprehensive histopathology, and systematic grading. Each liver was subjected to gross evaluation, assessing color, size, and texture, and grading the lesions on a 0–3 scale (0 = normal, 1 = mild, 2 = moderate, 3 = severe). Subsequently, the total sum of the grades for each sample was calculated. A specimen of 0.5 cm in thickness was taken and underwent fixation in 10% buffered formalin, followed by embedding in paraffin and sectioning at a thickness of 4 μm. Tissue sections were then stained using hematoxylin and eosin (HE), Silver Stain for reticular fibers detection (SS, Diapath kit, Code 010211, Diapath S.p.A, Martinengo, Italy), Masson’s Trichrome (MT, Diapath kit, Code 010210, Diapath S.p.A, Martinengo, Italy), and Weigert–Van Gieson (VG, Diapath kit, 010243, Diapath S.p.A, Martinengo, Italy) staining. The degree of hepatocellular vacuolization was evaluated with HE staining, while reticular stromal architecture was assessed using SS staining. Grading criteria and systems established by Trott et al. (2014) [5] were employed. The grading system of hepatocellular vacuolization was modified as follows: grade 1 corresponded to very rare vacuolization, grade 2 to less than 50% of hepatocytes containing vacuoles, and grade 3 to vacuolization in 50% or more of hepatocytes. Collagenous connective tissue content and vascular wall changes, including elastin alterations, were examined using MT and VG staining, respectively. Collagen connective tissue deposition was graded on a 0–3 scale (0 = no fibrosis, 1 = focal perivascular fibrosis, 2 = multifocal perivascular fibrosis and 3 = multifocal perivascular and intraparenchymal fibrosis based on Aziza et al. (2019) [34]. Following the methodology described by Trott et al. (2014) [5], elastin presence was evaluated in medium and large vessels using the VG staining. Additionally, immunohistochemical analysis was performed using the primary antibody TNF-alpha (NBP1-19532, Novus, Abingdon, UK). Following heat-induced (95–98 °C) antigen retrieval with citrate buffer pH 6.0 for 18 min, and hydrogen peroxidase block for 10 min (Ultravision Hydrogen Peroxide Block, Epredia, Breda, The Netherlands), the primary antibody was incubated for 60 min (dilution 1:100) and was labeled using the Ultravision Quanto Detection System HRP DAB detection kit, following the instructors guidelines (TL-060-QHD, Epredia, Breda, The Netherlands). IHC labeling was evaluated based on the cytoplasmic intensity of hepatocytes’ staining on the total surface area, scored on a scale from 1 to 4 (1 = 0–25%, 2 = 25–50%, 3 = 50–75%, 4 = 75–100%).

### 2.8. Statistical Analysis

Statistical analysis of data was performed with the use of SPSS software (SPSS 28.0 Version, Chicago, IL, USA). One-way ANOVA was employed to analyze the effects of treatments on the tested variables and Tukey’s test for the post hoc evaluation. Statistical difference was set at *p* < 0.05, and results were presented as average values ± standard deviation (SD). The effect of treatments on major fatty acid groups was analyzed with ANOVA and post hoc Tukey’s test with the use of GraphPad Prism (version 10.2.3 for Windows^®^, GraphPad Software, San Diego, CA, USA). Statistical evaluation of the score results of liver gross evaluation, histopathology and immunohistochemistry was performed by Chi-squared tests applied with SPSS (expressed as Asymptotic Significance-X^2^Value) and the results are presented in bar graphs as the relative frequency in percentage of scores for each parameter separately and for each treatment with the use of GraphPad Prism (version 10.2.3 for Windows^®^, GraphPad Software, San Diego, CA, USA). The statistical unit for the evaluation of performance parameters was the pen (replicate), while the statistical unit for the evaluation of the rest parameters was the sample grouped within its treatment. The investigation of correlations between scoring variables describing the liver gross evaluation, histopathology and immunohistochemistry results was done with Spearman’s correlation test in SPSS software.

## 3. Results

### 3.1. Performance of Laying Hens

**Figure 1:**
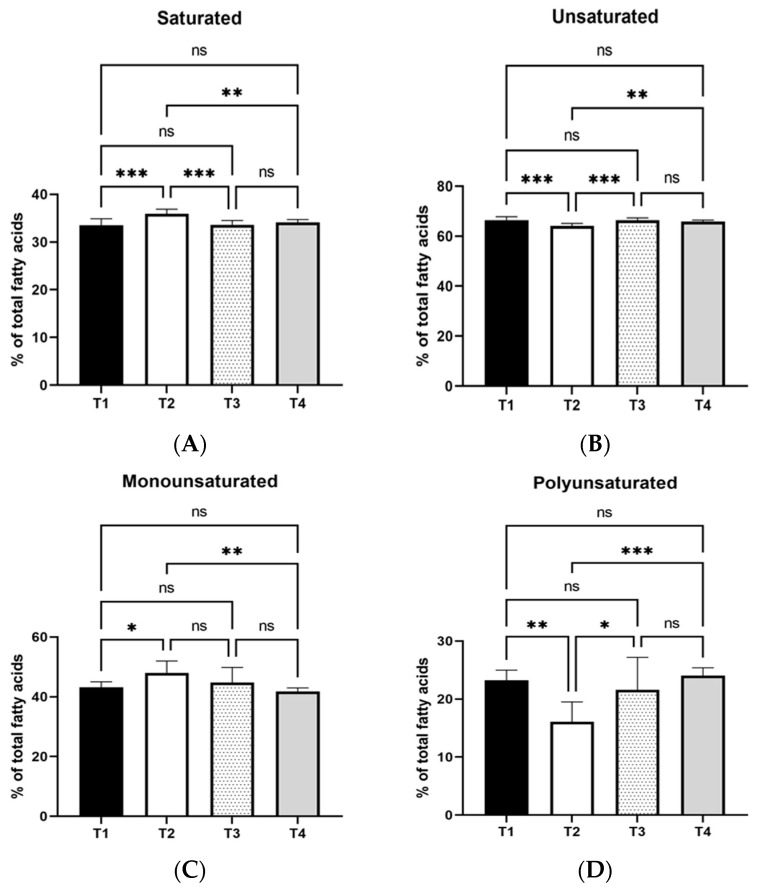
Effects of three levels of dietary MT extract on saturated egg yolk (**A**), unsaturated (**B**), monounsaturated (**C**), and polyunsaturated (**D**) fatty acid percentage. T1: control; T2: basal diet with 1% MT extract; T3: basal diet with 2.5% MT extract; T4: basal diet with 4% MT extract. *: mean values differ significantly between them (*p* < 0.05); **: mean values differ significantly between them (*p* < 0.01); ***: mean values differ significantly between them (*p* < 0.001); ns: not significant (*n* = 8).

The dietary supplementation of silymarin did not affect the mean values of the performance parameters of the treated groups (Table 2). However, there is a tendency for gradually higher feed intake as the supplementation rate increased (Table 2; Figure 1). The only significant effect was reported on egg production (%) in week 7, with T2 and T3 demonstrating higher values compared to the control group, although no difference among the treatments was mentioned until this week.

### 3.2. Egg Quality Characteristics

The results for the 1st, 4th, and 8th week, along with the overall experimental period are presented in Table 3. Based on the overall experimental period, the addition of silymarin was linked with somewhat compromised egg weight compared to the control group, especially for T3 (*p* = 0.011), while this effect was also present on the albumen weight (*p* = 0.001). Moreover, T2 illustrated lower Haugh units compared to all other treatments (*p* = 0.002). Regarding egg yolk color, T3 and T4 presented darker yolks (*p* < 0.001) with higher a* values (*p* < 0.001), compared to T1 and T2, while only T3 showed also higher b* values (*p* = 0.056) than T1 and T2. On the other hand, both T3 and T4 illustrated somewhat decreased L* values compared to T2. At the end of the first week almost all parameters did not show any significant difference, apart from the yolk color, which was almost darker for T4 compared to all other treatments, especially to T2 (*p* = 0.027). The same effect was also obvious on a* values with the T3 and the T4 values being higher than the T2 ones (<0.001). At the end of week 4, changes in the egg weight became significant, with the eggs of T3 being lighter than the eggs of T2 (*p* = 0.011). Similar effects were apparent regarding the albumen weight, which was lighter for T3 compared to T2 and T1 as well (*p* = 0.008). Moreover, there were important changes in the egg shape as well, with the T3 eggs presenting a shorter longitudinal axis than the T2 eggs (*p* = 0.004) and a shorter transverse axis than the eggs of the control group (*p* = 0.023). Also, T2 presented lower Haugh units than all other treatments (*p* < 0.001). With regards to the yolk color, treatments T3 and T4 showed darker yolks compared to both the control group and T2 (*p* = 0.001). The same effect was also apparent in the a* values (*p* < 0.001). At the end of week 8, T3 eggs had lighter albumen weight compared to T1 (*p* = 0.015). The yolk color was darker for T3 and T4 (*p* = 0.001), while the same held for a* and b* values (*p* < 0.001; *p* = 0.01). On other hand, T2 presented greater L*values than T3 and T4 (*p* = 0.014).

### 3.3. Egg Yolk Oxidation, Total Phenol Content (TPC)

MDA levels in yolk samples were significantly lower in T2 group, whereas there was no statistical difference among the other treatments (*p* = 0.029) (Table 4). On the other hand, the egg yolk TPC expressed as µg of Gallic Acid equivalents per gram of dry yolk (µg GAE/g), presented no significant difference among the treatments.

### 3.4. Egg Yolk Fatty Acid Profile

The analysis of egg yolk fatty acids showed that several individual fatty acids were affected by the addition of the extract in the feed. According to Table 5, treatment T2 enhanced the portion of Myristic (14:0), Myristoleic (14:1n5), Palmitic (16:0), Palmitoleic (16:1n7), Elaidic (18:1n9t), Oleic (18:1n9c), cis-Vaccenic (18:1n7c), α-Linoleic (18:3n3) and all-cis-6,9,12,15-Octadecatetraenoic (18:4n3) fatty acids compared not only to the control group but also to T3 and T4 (*p* = 0.0075; *p* < 0.001; *p* = 0.005; *p* = 0.003; *p* < 0.001; *p* = 0.024; *p* = 0.005; *p* = 0.018; *p* = 0.009). On the contrary, T2 limited the ratio of cis-7-Hexadecenoic (16:1n9), Margaric (17:0), Linelaidic (18:2n6t), Linoleic (18:2n6c) and cis, cis-11,14-Eicosadienoic (20:2n6) fatty acids compared to the other groups (*p* = 0.041; *p* = 0.033; *p* = 0.077; *p* < 0.001; *p* = 0.004). Finally, T4 showed the highest proportion of Arachidic (20:0) and γ-Linoleic (18:3n6) acids (*p* = 0.014; *p* < 0.001).

**Figure 2:**
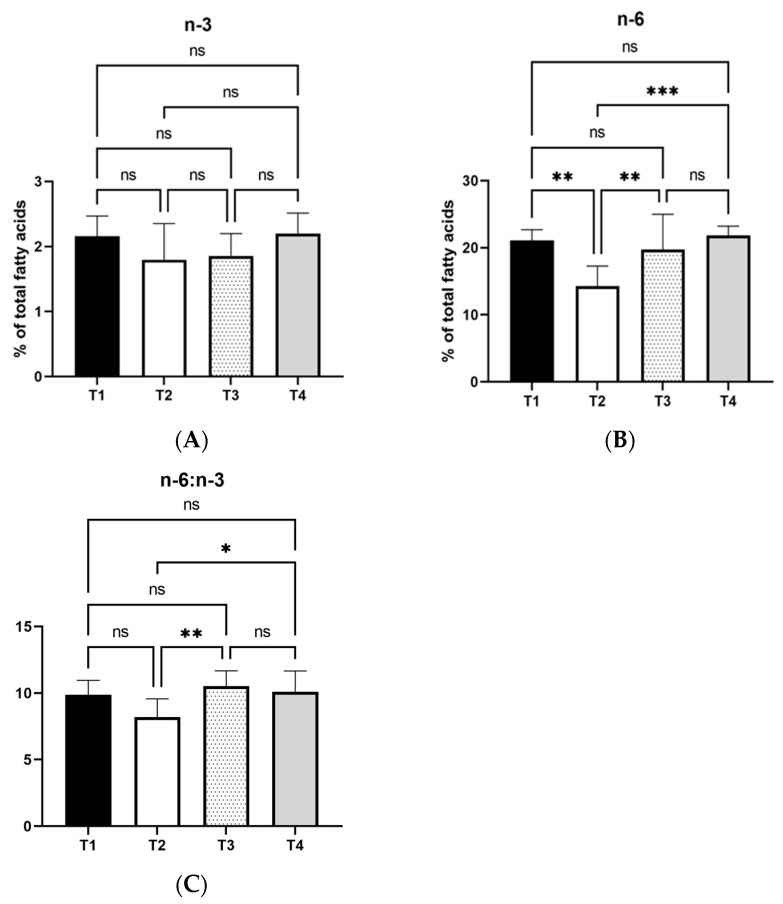
Effects of three levels of dietary MT extract on egg yolk on n-3 (**A**), n-6 (**B**), and n-6:n-3 ratio (**C**). T1: control; T2: basal diet with 1% MT extract; T3: basal diet with 2.5% MT extract; T4: basal diet with 4% MT extract. *: mean values differ significantly between them (*p* < 0.05); **: mean values differ significantly between them (*p* < 0.01); ***: mean values differ significantly between them (*p* < 0.001); ns: not significant (*n* = 8).

Regarding the major fatty acid categories, the statistical analysis results in Figure 1 and Figure 2 illustrated that T2 increased the saturated fatty acids compared to T1, T3, and T4 (*p* < 0.001; *p* < 0.001; *p* < 0.01). On the contrary, T2 decreased the unsaturated fatty acids relative to T1, T3, and T4 (*p* < 0.001; *p* < 0.001; *p* < 0.01). Moreover, the monounsaturated fatty acid (MUFA) portion was lower in T1 (*p* < 0.05) and T4 (*p* < 0.01) relative to T2, whereas the exact opposite held in the polyunsaturated (PUFA) fatty acids that were higher in T1, T4 and T3 (*p* < 0.01; *p* < 0.001; *p* < 0.05) compared to T2. No differences were detected in n-3 fatty acids’ proportions among the treatments. However, the T2 restricted the n-6 fatty acids’ percentages relative to T1, T3 and T4. (*p* < 0.01; *p* < 0.01; *p* < 0.001). Finally, the ratio of n-6:n-3 was higher in T3 (*p* < 0.01) and T4 (*p* < 0.05) compared to T2.

### 3.5. Gross Evaluation, Histopathology and Immunohistochemistry

The effect of dietary treatments on the macroscopical evaluation of the color, the size, the texture and the sum of the macroscopical scoring is presented in Table 6. Macroscopically, there was an overall trend towards a difference in total macro-scoring between treatments (*p* = 0.059) (Table 6). Specifically, the total observations of T2 and T3 were allocated in a narrow scoring scale between 0 and 2, whereas T1 and T4 showed a trend towards a somewhat wider deviation. This particular trend was mainly attributed to the difference in color (x^2^ = 0.013), given that both the texture and the size were similar among the treatments for the tested samples (Table 6).

**Figure 3:**
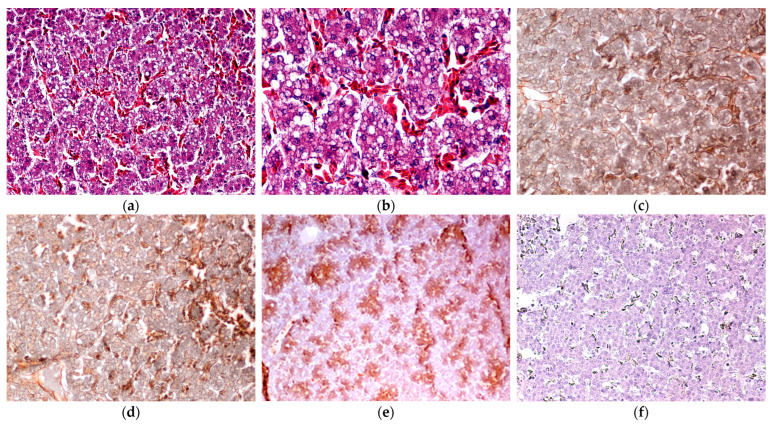
Liver, chicken. (**a**) Hepatocellular vacuolization of more than 50% of the parenchyma characterized by variably sized vacuoles (grade 2). Hematoxylin and eosin, ×20. (**b**) Higher magnification of the (**a**). Hematoxylin and eosin, ×40. (**c**) Normal reticulin lines demonstrate the normal architecture of hepatic plates, and (**d**) focal loss of sinusoidal reticulin visibility in an area characterized by more extensive vacuolar change. Silver Stain, ×20. (**e**) TNF-alpha immunohistochemically labeled hepatocytes; note the group of cells showing strong immunoreactivity, compared to the other hepatocytes, (**f**) control slide lacking the primary antibody. IHC: diaminobenzidine chromogen, hematoxylin counterstain, ×10.

**Figure 4:**
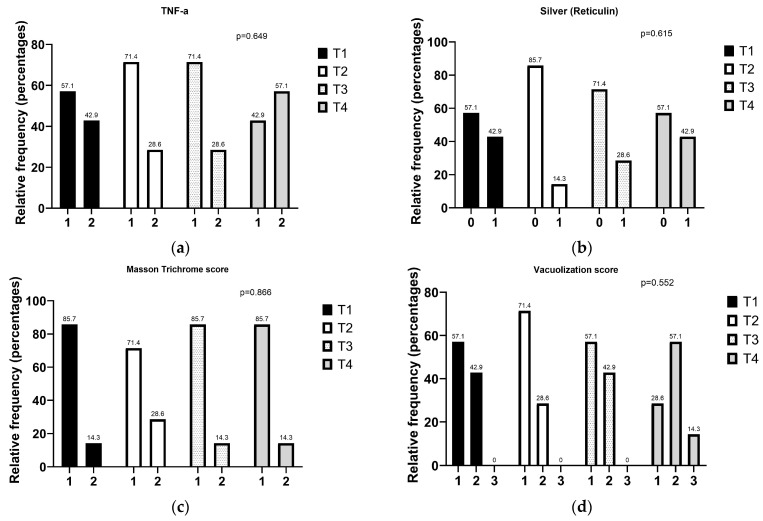
Effect of dietary treatments (T1, T2, T3, T4) on (**a**) the relative frequency of TNF-alpha immunohistochemically labeled hepatocytes (1 = 0–25% of the cells in total surface area showed intense cytoplasmic reaction, 2 = 25–50% of the cells in total surface area showed intense cytoplasmic reaction), (**b**) silver stain reticulin distribution architecture (0 = normal distribution, 1= focal areas of inferior visibility), (**c**) perivascular–Masson’s Trichrome labeled connective tissue (1 = focal perivascular fibrosis, 2 = multifocal perivascular fibrosis), and (**d**) hepatocellular vacuolization score (1 = very rare vacuolization, 2 = less than 50% containing vacuoles, 3 = 50% or greater containing vacuoles).

No group exhibited severe lesions. A significant difference was found in the evaluation of liver discoloration (*p* = 0.013). Groups T2 and T3 showed milder lesions, with statistically significant differences observed between group T3 and both groups T1 and T4. Microscopical findings are summarized in Figure 3 and Figure 4. The hepatic vacuolization assessed in HE sections ranged from mild to moderate across all groups (Figure 3 and Figure 4). Group T2 exhibited the lowest degree of hepatic vacuolization, followed by groups T3 and T1. The reticulin distribution architecture showed only focal areas of inferior visibility, which were correlated with the degree of hepatic vacuolization (r = 0.751, *p* < 0.001) and gross discoloration (r = 0.393, *p* = 0.038). Fibrotic changes observed with MT staining were mild and showed no statistical significance among the groups. Additionally, no differences were found in the elastin and elastic laminae, as viewed with VG staining. TNF-α staining evaluation was limited to grade 2 of the followed grading system, with no significant differences among the groups. Interestingly, TNF-α staining demonstrated a positive correlation with the total macro-scoring and individually with the color and the texture grading (r = 0.649, *p* < 0.001; r = 0.590, *p* < 0.001; and r = 0.666, *p* < 0.001, respectively).

## 4. Discussion

The calculated total choline content of the diets was close to the requirements that are indicated by the ISA brown management guide. Nevertheless, it is generally acknowledged that choline bioavailability from cereal grains is compromised and is not totally available. For this reason, choline chloride dietary supplementation is a regular practice in the poultry industry and is essential for laying hens’ diets. Choline regulates lipid metabolism in laying hens’ livers and contributes to antioxidant mechanisms. Choline, a methyl donor, is needed for hepatic VLDL secretion, which induces fat accumulation [35]. After four weeks of supplementation, ISA hens that were fed high-fat, cholesterol, and low-choline diets developed hepatic steatosis [36]. Choline supplementation in laying hens boosted GSH-Px and T-AOC activity and decreased liver MDA [37]. Thus, silymarin in any form has only been studied in diets with adequate choline. For this reason, we used diets lacking choline chloride supplementation to investigate silymarin’s potential benefit without a choline chloride synergistic effect. Silibinin inhibited TNF-a gene expression in the liver of choline-deficient mice, reducing hepatocellular vacuolation and lobular inflammation [38]. The experiment’s diets contained similar protein and energy content. A diet with up to 6% extra saturated fat was supplied in laying hens with protein–energy imbalances to produce fatty liver hemorrhagic syndrome [39]. In the current study, fat supplementation was mostly polyunsaturated fats and did not exceed 2.7% (T4 group maximum). The absence of significant performance impacts compared to other research suggests that post-peak laying period diets should be supplemented with choline chloride. However, improvements in egg quality indicators crucial to the egg industry, such as yolk color, may suggest that MT extract could be an additional dietary element to maintain efficient lipid metabolism in laying hens.

Dietary silymarin may improve laying hen performance indicators such the egg-laying rate, feed intake (FI), and feed conversion ratio (FCR) [29,40]. Improved feed consumption was attributed to higher jejunal villus height. In another study, Šťastník et al. (2019) [41] found that egg production rose, but FI did not affect FCR. Kavan et al. (2023) [27] reported similar results with 0.2 g/kg milk thistle inclusion. Different performance impacts were discovered in this study. Prior research did not employ diets lacking choline chloride supplementation; thus, it is possible that MT extract supplementation did not have a significant performance effect in our setting.

According to previous findings, egg weight and Haugh units affected egg quality. Quarantelli et al. (2009) [29], Kavan et al. (2023) [27], and Faryadi et al. (2021) [30] found that silymarin-fed groups had heavier eggs. MT supplementation did not increase egg weight compared to the control group in this study. Previous investigations revealed silymarin supplementation enhanced Haugh units [27,30,41]. The Haugh unit is mostly affected by egg ovomucine content, not freshness [42,43,44]. Silymarin may raise the Haugh unit by promoting liver cell renewal and nutrition digestion [12,45,46]. In the current investigation, MT extract at 1% decreased Haugh units, but higher levels did not vary from the control group. Free radicals and oxidative damage may damage egg proteins, hence the T2 (1% MT) group may have a larger oxidative effect [47]. Since MT extract lacks choline, supplementation at 1% may not protect egg quality indicators influenced by oxidative processes. Oxidative stress degrades egg white protein, lowering albumen viscosity and HU [48].

Carotenoids are deposited in the egg yolk after enzymatic changes in the hen. These alterations include oxidation and cleavage [49]. Egg yolk color depends on carotenoids deposition or metabolism [50]. Due to its antioxidant capabilities, silymarin may increase yolk carotenoids or related substances while protecting yolk pigments from oxidative damage [51]. Micelle silymarin supplementation increased egg yolk color linearly over 8 weeks in a recent study. In the current investigation, MT extract supplementation significantly affected egg yolk color except at 1%. Except for the yolk color index, silymarin supplementation has not been studied on egg yolk color parameters. We report L*, a*, and b* levels here to provide more proof. In particular, the two higher levels of MT extract supplementation raised redness (a*) substantially compared to the lower level and control treatment. The dosage response association between lutein supplementation in diets and egg yolk content showed that higher lutein content increased egg yolk redness [52]. Thus, the 1% inclusion rate (T2) may have reduced yolk carotenoid concentration or modified its profile. It may also explain this group’s greater lightness (L*). An exact mechanism for this impact is unknown. Given the diets lacking choline chloride supplementation and the low MDA concentration of the T2 egg yolk, carotenoids may have been considerably stimulated and spared to retain an efficient antioxidant capability. Additionally, the reduced carotenoid levels, as indicated by egg yolk color, may be linked to the lower ratio of dietary unsaturated to saturated fatty acids in T2 relative to the other treatments [53]. Feeding laying hens’ diets with a more saturated profile, as observed in the T3 and T4 groups, may result in reduced carotenoid content in the egg yolk [54]. Additional evidence for our findings is presented by Cherian et al. (2007) and Cherian (2008) [55,56], who observed an increased yolk color index following dietary supplementation with omega-3 fatty acids, including fish oil. In the current study, the redness of egg yolk color has been enhanced due to MT supplementation. However, the current study did not evaluate carotenoid levels and their profiles, indicating a need for further research in this direction.

Regarding egg yolk lipid oxidation, the 1% group (T2) had the lowest MDA value, whereas the 2.5% and 4% were significantly higher and similar to the control group. MDA is a lipid oxidation product used to evaluate lipid peroxidation [57]. Based on diet formulation, each treatment gradually raised extract inclusion and soybean oil content. Thus, T3 and T4 MDA measurements should be substantially higher than the other groups. Supplementation of alternate lipid sources and milk thistle meal increased oxidative stability, suggesting that polyunsaturated fatty acid diets may need more natural antioxidants [58]. Regardless of concentration or form, silymarin did not affect the MDA content of fresh eggs or eggs held for 28 days at room temperature or in the fridge [30]. In this study, T3 and T4 had similar MDA yolk concentration, likely because of the extract’s antioxidant properties. Compared to T3 and T4, which did not differ and were similar to the control group in both parameters, the 1% inclusion rate of MT extract increased saturated fatty acids and decreased unsaturated ones. The decrease in unsaturated fatty acids was ascribed to the restriction of polyunsaturated fatty acids, despite T2 exhibiting the highest proportion of monounsaturated fatty acids. The type of dietary fatty acids influences the fatty acid profile of egg yolk [59]. It can be hypothesized that the variation in the fatty acid profile of T2 is primarily attributable to the associated diet. The elevated levels of unsaturated fatty acids in the T3 and T4 groups may indicate the antioxidant capacity of the MT extract, even in diets lacking choline chloride supplementation. This field warrants further investigation.

A histology investigation was performed to better understand how the extract affects liver health. Hepatic vacuolar alterations were compared to the liver’s gross appearance, reticulin integrity, connective tissue, collagen, and vascular elastin. Trott et al. (2014) suggested a grading system [5] that correlates positively with Oil Red O (ORO) staining, which particularly identifies lipid content, for a more accurate assessment. Although no positive correlation was found between macroscopic lesions and microscopic vacuolization grading, gross discoloration was positively correlated with reticulin distribution changes, suggesting that this correlation may indicate vacuolization severity and architecture changes. A recent study reported that mulberry leaf extract reversed liver damage in high-energy–low-protein laying chickens [39]. The extract reduced liver cell vacuolar degeneration and gradually returned them to their normal healthy state [39]. In the current study, no significant differences were seen in hepatic vacuolization scoring across treatments, but MT extract supplementation may have maintained a similarly low level as the control group. In the control group, 1% oil source was added, whereas in T3 and T4, it was 1.83% and 2.7%. While prior research suggests that silybin, an active component in milk thistle, influences TNF-α liver expression, this study showed no significant differences among groups. Treatments were given to normal hens without experimental liver fatty composition intervention, which may explain the lack of change [38]. The association between TNF-α staining and overall macro-scoring, and individually with color and texture grading, may indicate that liver appearance could reflect both microscopic and biochemical alterations in the liver. However, it is important to note that this finding comes with certain limitations, and further broader and more targeted studies are required to confirm and better elucidate this correlation.

## 5. Conclusions

In summary, dietary supplementation of MT extract in diets lacking choline chloride supplementation may influence certain egg quality indices. In particular, the dietary MT extract could affect the egg yolk color, mainly by increasing the redness (a* value). Moreover, the extract could maintain low MDA levels and high contents of unsaturated fatty acids in the egg yolks of the treated layers. These results may suggest that MT extract supplementation could safeguard hepatic health and may enhance egg quality in hens’ fed diets lacking choline chloride supplementation. Further research is needed towards investigating the potential synergistic effects of choline chloride exogenous supplementation with the MT extract used in the present study on the examined parameters with emphasis placed on liver pathological alterations.

## Figures and Tables

**Table 1:** Ingredients and nutrients of dietary treatments fed to laying hens during the experimental period. The calculated nutrient content of total choline (mg/kg of diet) is based on the total choline content of feedstuffs used in the diets. All diets lacked choline chloride supplementation.

Ingredients (%)	Treatments			
	T1	T2	T3	T4
Wheat soft	4.89	4.00	0.00	0.00
Corn	58.22	57.16	59.52	56.76
Soybean meal (47% CP)	20.46	20.77	19.78	19.96
Lucerne meal	3.00	2.75	2.00	2.00
Soybean oil	1.00	0.61	1.63	2.50
Palm fat	0.00	1.00	0.20	0.20
Gluten 60%	1.03	1.01	2.20	2.48
Sunflower meal (29% CP)	1.30	1.65	2.12	2.04
Vitamin premix	0.20	0.20	0.20	0.20
Mineral premix	0.20	0.20	0.20	0.20
Sodium chloride	0.19	0.23	0.23	0.24
Lysine HCL	0.00	0.00	0.01	0.00
DL-Methionine	0.13	0.12	0.12	0.12
limestone	8.75	8.76	8.75	8.74
sodium sulphate	0.22	0.11	0.11	0.11
Yeast	0.10	0.10	0.10	0.10
Lysolecithin	0.05	0.05	0.05	0.05
monocalciumphosphate	0.25	0.25	0.27	0.30
Phytase	0.01	0.01	0.01	0.01
Milk thistle extract (MT)	0.00	1.00	2.50	4.00
Calculated Analysis				
Crude protein (%)	16.5	16.5	16.5	16.5
Crude fiber (%)	3.20	3.20	3.07	3.00
Crude fat (%)	4.00	4.00	4.81	5.57
Crude ash (%)	12.09	12.09	11.94	11.93
Total Choline (mg/kg)	1021.7	1021.4	988.2	980.6
Metabolizable Energy (kcal/kg)	2726	2726	2723	2723

Premix composition. Provided per kg diet: Retinyl acetate: 4.2 mg; Cholecalciferol: 0.1 mg; tocopherol acetate: 31.25 mg; Menadione: 5.0 mg; Cyanocobalamin: 0.025 mg; folic acid: 1.0 mg; Pantothenic acid: 12.5 mg; Riboflavin, 6.25 mg; Nicotinic acid: 43.75 mg; Thiamin: 3.0 mg; D-biotin: 0.1 mg; Pyridoxine: 5.0 mg; Manganese: 125 mg; Zinc: 112 mg; Iron: 62 mg; Copper: 10 mg; Iodine: 1.0 mg; Selenium: 0.15 mg.

**Table 2:** Effect of dietary treatments (T1, T2, T3, T4) on performance parameters (egg weight; egg production in %; egg mass; feed intake per hen daily; feed conversion ratio—FCR) of laying hens throughout the experimental period. Results are presented as mean values ± standard deviation (*n* = 15 per treatment).

Parameters	Treatments				*p*-Value
	T1	T2	T3	T4	
**Week 1**					
Egg weight (g)	62.81 ± 3.27	61.96 ± 1.61	62.09 ± 2.96	63.56 ± 2.74	0.880
Egg Production (%)	92.04 ± 3.27	92.68 ± 6.93	91.53 ± 4.83	83.12 ± 1.29	0.101
Egg Mass	57.80 ± 3.41	57.39 ± 3.63	56.83 ± 4.06	52.82 ± 1.96	0.312
Feed/Hen/Day	123.25 ± 3.88	117.34 ± 5.97	125.05 ± 8.11	123.28 ± 5.53	0.472
FCR	2.14 ± 0.11	2.05 ± 0.22	2.20 ± 0.03	2.33 ± 0.08	0.137
**Week 2**					
Egg weight (g)	61.92 ± 4.85	62.95 ± 0.94	65.96 ± 0.37	62.29 ± 1.76	0.295
Egg Production (%)	87.61 ± 4.69	92.10 ± 6.76	89.55 ± 5.13	87.24 ± 6.12	0.722
Egg Mass	54.27 ± 5.49	57.94 ± 3.45	59.06 ± 3.14	54.27 ± 2.35	0.342
Feed/Hen/Day	117.94 ± 2.58	119.11 ± 3.67	121.88 ± 7.36	121.99 ± 7.36	0.767
FCR	2.19 ± 0.26	2.06 ± 0.11	2.07 ± 0.14	2.25 ± 0.24	0.569
**Week 3**					
Egg weight (g)	63.44 ± 2.52	60.64 ± 2.92	62.81 ± 1.10	64.36 ± 1.39	0.250
Egg Production (%)	88.42 ± 3.60	88.00 ± 4.54	91.17 ± 2.42	88.35 ± 2.17	0.641
Egg Mass	56.13 ± 4.00	53.28 ± 0.60	57.27 ± 2.18	56.85 ± 0.88	0.229
Feed/Hen/Day	116.72 ± 3.81	118.61 ± 6.12	120.84 ± 5.44	117.69 ± 4.93	0.789
FCR	2.08 ± 0.12	2.23 ± 0.12	2.11 ± 0.04	2.07 ± 0.08	0.249
**Week 4**					
Egg weight (g)	64.52 ± 3.58	64.44 ± 4.10	60.77 ± 1.11	63.11 ± 3.78	0.521
Egg Production (%)	84.69 ± 3.69	86.32 ± 3.00	87.16 ± 5.35	89.92 ± 2.62	0.447
Egg Mass	54.64 ± 3.83	55.59 ± 3.29	52.93 ± 2.55	56.81 ± 5.02	0.654
Feed/Hen/Day	117.99 ± 6.50	123.85 ± 0.70	120.82 ± 6.27	121.91 ± 4.72	0.585
FCR	2.16 ± 0.13	2.23 ± 0.13	2.29 ± 0.16	2.15 ± 0.11	0.598
**Week 5**					
Egg weight (g)	63.40 ± 4.32	61.85 ± 2.83	56.31 ± 9.42	62.12 ± 1.19	0.435
Egg Production (%)	86.97 ± 7.38	87.88 ± 4.14	86.92 ± 1.23	89.43 ± 1.59	0.881
Egg Mass	55.19 ± 6.74	54.40 ± 4.59	48.95 ± 8.23	55.56 ± 1.80	0.513
Feed/Hen/Day	116.60 ± 6.66	122.44 ± 5.45	119.84 ± 6.62	121.49 ± 9.02	0.760
FCR	2.13 ± 0.24	2.27 ± 0.28	2.49 ± 0.44	2.19 ± 0.15	0.495
**Week 6**					
Egg weight (g)	62.32 ± 2.10	62.46 ± 2.35	60.45 ± 5.47	61.25 ± 2.06	0.862
Egg Production (%)	88.74 ± 1.43	87.72 ± 1.18	87.84 ± 2.09	86.48 ± 5.38	0.838
Egg Mass	55.32 ± 2.75	54.80 ± 2.48	53.06 ± 4.48	52.94 ± 2.94	0.749
Feed/Hen/Day	112.55 ± 9.82	121.88 ± 6.86	121.21 ± 4.35	121.32 ± 4.36	0.333
FCR	2.03 ± 0.12	2.22 ± 0.06	2.29 ± 0.15	2.30 ± 0.14	0.089
**Week 7**					
Egg weight (g)	63.82 ± 1.20	65.16 ± 2.49	62.57 ± 7.17	64.77 ± 2.09	0.858
Egg Production (%)	83.46 ± 0.89 ^a^	89.99 ± 1.35 ^b^	89.18 ± 3.55 ^b^	86.70 ± 0.78 ^ab^	0.015
Egg Mass	53.27 ± 1.47	58.63 ± 2.20	55.69 ± 5.24	56.17 ± 2.24	0.295
Feed/Hen/Day	114.00 ± 3.32	118.40 ± 11.01	116.94 ± 8.35	122.00 ± 7.73	0.688
FCR	2.14 ± 0.12	2.02 ± 0.11	2.12 ± 0.29	2.18 ± 0.23	0.751
**Week 8**					
Egg weight (g)	61.34 ± 3.13	63.76 ± 4.43	63.11 ± 3.34	60.49 ± 2.87	0.652
Egg Production (%)	84.79 ± 9.75	82.33 ± 12.88	87.79 ± 3.88	90.29 ± 6.95	0.727
Egg Mass	51.83 ± 4.04	52.31 ± 7.42	55.47 ± 4.99	54.58 ± 4.28	0.809
Feed/Hen/Day	119.00 ± 3.77	125.88 ± 6.82	120.62 ± 2.84	119.61 ± 8.01	0.486
FCR	2.31 ± 0.25	2.45 ± 0.49	2.19 ± 0.18	2.20 ± 0.18	0.688

T1: control; T2: basal diet with 1% milk thistle (MT) extract; T3: basal diet with 2.5% MT extract; T4: basal diet with 4% MT extract. Values are means ± SD. Values in the same row with different superscripts differ significantly (*p* < 0.05).

**Table 3:** Effect of dietary treatments (T1, T2, T3, T4) on egg quality parameters on the 1st, 4th, and 8th week of the experiment and for the overall experimental period (*n* = 12 per treatment and per sampling week; *n* = 48 for the overall period).

Parameters	Treatments				*p*-Value
	T1	T2	T3	T4	
**1st week**					
Egg weight (g)	64.04 ± 4.60	59.55 ± 1.49	61.50 ± 5.41	62.75 ± 2.11	0.273
Yolk weight (g)	16.10 ± 1.50	16.13 ± 0.66	16.29 ± 1.38	16.6 ± 1.52	0.772
Albumen weight (g)	43.05 ± 3.53	39.04 ± 4.34	40.60 ± 4.23	41.80 ± 5.75	0.179
Eggshell weight (g)	4.90 ± 0.53	4.38 ± 1.01	4.63 ± 0.58	4.35 ± 0.58	0.205
Eggshell thickness (mm)	0.34 ± 0.55	0.30 ± 0.07	0.31 ± 0.03	0.30 ± 0.04	0.074
Longitudinal axis (mm)	56.56 ± 1.41	55.76 ± 1.76	56.65 ± 1.76	56.89 ± 2.35	0.477
Transverse axis (mm)	44.38 ± 1.19	44.04 ± 1.21	44.60 ± 1.91	45.32 ± 1.72	0.236
Shape index	0.78 ± 0.02	0.79 ± 0.16	0.79 ± 0.03	0.80 ± 0.21	0.566
Eggshell color	18.94 ± 2.91	18.78 ± 4.35	17.50 ± 2.22	18.13 ± 2.48	0.314
Yolk color fan score	8.83 ± 0.72 ^ab^	8.50 ± 1.31 ^a^	8.92 ± 0.67 ^ab^	9.67 ± 0.89 ^b^	0.027
Haugh units	91.50 ± 6.71	87.50 ± 13.91	90.17 ± 4.53	88.83 ± 5.17	0.686
L*	69.65 ± 4.00	69.66 ± 3.91	71.55 ± 3.30	68.08 ± 5.82	0.286
a*	9.36 ± 1.55 ^ab^	7.60 ± 1.87 ^a^	10.41 ± 1.62 ^bc^	11.83 ± 2.97 ^c^	<0.001
b*	53.30 ± 7.33	52.98 ± 10.34	57.78 ± 4.96	54.12 ± 6.33	0.391
**4th week**					
Egg weight (g)	64.61 ± 5.44 ^ab^	66.93 ± 5.35 ^a^	58.32 ± 8.00 ^b^	62.46 ± 5.40 ^ab^	0.011
Yolk weight (g)	16.98 ± 1.02	17.96 ± 1.73	16.48 ± 1.69	17.64 ± 1.44	0.092
Albumen weight (g)	43.35 ± 4.70 ^a^	44.05 ± 4.15 ^a^	37.51 ± 6.10 ^b^	40.20 ± 3.99 ^ab^	0.008
Eggshell weight (g)	4.28 ± 0.53	4.93 ± 0.61	4.33 ± 0.65	4.61 ± 1.03	0.111
Eggshell thickness (mm)	0.30 ± 0.04	0.33 ± 0.06	0.30 ± 0.02	0.31 ± 0.06	0.065
Longitudinal axis (mm)	56.48 ± 1.79 ^ab^	58.76 ± 1.98 ^a^	53.83 ± 5.14 ^b^	57.33 ± 1.94 ^ab^	0.004
Transverse axis (mm)	45.69 ± 1.19 ^a^	45.56 ± 1.32 ^ab^	42.89 ± 4.35 ^b^	45.66 ± 1.26 ^ab^	0.023
Shape index	0.80 ± 0.02	0.78 ± 0.15	0.80 ± 0.10	0.80 ± 0.02	0.455
Eggshell color	15.44 ± 2.37	16.34 ± 3.78	17.40 ± 2.91	17.28 ± 2.57	0.373
Yolk color fan score	9.33 ± 0.78 ^a^	9.50 ± 0.52 ^ab^	10.17 ± 0.84 ^bc^	11 ± 0.87 ^c^	0.001
Haugh units	95.50 ± 5.67 ^a^	85.17 ± 6.42 ^b^	95.33 ± 6.05 ^a^	96.44 ± 5.01 ^a^	<0.001
L*	71.97 ± 2.04	74.73 ± 1.41	68.02 ± 11.18	71.40 ± 2.06	0.071
a*	9.30 ± 1.64 ^a^	8.55 ± 1.32 ^a^	10.29 ± 1.83 ^ab^	12.23 ± 2.20 ^b^	<0.001
b*	56.68 ± 4.05	58.60 ± 4.47	55.66 ± 6.76	58.64 ± 4.66	0.434
**8th week**					
Egg weight (g)	61.62 ± 5.40	58.98 ± 6.40	54.71 ± 7.05	57.25 ± 9.08	0.128
Yolk weight (g)	16.35 ± 1.71	16.18 ± 1.62	16.91 ± 1.94	17.16 ± 1.70	0.477
Albumen weight (g)	40.80 ± 4.92 ^a^	38.21 ± 5.01 ^ab^	33.18 ± 5.49 ^b^	35.78 ± 7.21 ^ab^	0.015
Eggshell weight (g)	4.48 ± 0.67	4.58 ± 0.78	4.62 ± 0.88	4.31 ± 0.82	0.776
Eggshell thickness (mm)	0.31 ± 0.05	0.31 ± 0.06	0.32 ± 0.06	0.33 ± 0.06	0.685
Longitudinal axis (mm)	57.100 ± 1.56	56.79 ± 2.00	56.77 ± 1.82	57.60 ± 3.12	0.771
Transverse axis (mm)	45.43 ± 1.51	44.73 ± 1.63	44.76 ± 1.64	44.89 ± 2.44	0.772
Shape index	0.80 ± 0.32	0.79 ± 0.02	0.79 ± 0.02	0.78 ± 0.32	0.528
Eggshell color	16.50 ± 2.11	17.61 ± 3.98	18.88 ± 3.64	15.86 ± 1.94	0.950
Yolk color fan score	10.17 ± 0.58 ^ab^	9.83 ± 0.39 ^a^	10.67 ± 0.49 ^bc^	10.83 ± 0.84 ^c^	0.001
Haugh units	92.50 ± 6.43	88.33 ± 7.22	89.42 ± 8.62	93.83 ± 5.25	0.196
L*	71.07 ± 2.97 ^ab^	72.86 ± 2.42 ^a^	69.57 ± 2.67 ^b^	69.85 ± 2.35 ^b^	0.014
a*	9.52 ± 0.74 ^a^	8.05 ± 1.74 ^a^	13.72 ± 2.51 ^b^	12.67 ± 2.09 ^b^	<0.001
b*	56.58 ± 5.31 ^a^	58.00 ± 4.17 ^a^	65.54 ± 7.45 ^b^	60.62 ± 3.05 ^ab^	0.010
**Overall period**					
Egg weight (g)	63.42 ± 5.18 ^a^	61.82 ± 6.61 ^ab^	58.18 ± 7.27 ^b^	60.67 ± 7.81 ^ab^	0.011
Yolk weight (g)	16.48 ± 1.44	16.75 ± 1.63	16.55 ± 1.66	17.09 ± 1.58	0.381
Albumen weight (g)	42.40 ± 4.45 ^a^	40.44 ± 5.10 ^ab^	37.10 ± 6.03 ^b^	39.18 ± 6.35 ^ab^	0.001
Eggshell weight (g)	4.55 ± 0.62	4.63 ± 0.82	4.52 ± 0.71	4.41 ± 0.80	0.655
Eggshell thickness (mm)	0.31 ± 0.05	0.31 ± 0.06	0.31 ± 0.04	0.31 ± 0.05	0.999
Longitudinal axis (mm)	56.71 ± 1.57	57.10 ± 2.25	55.75 ± 3.50	57.27 ± 2.50	0.061
Transverse axis (mm)	45.17 ± 1.39	44.78 ± 1.50	44.08 ± 2.95	45.26 ± 1.89	0.067
Shape index	0.80 ± 0.26	0.78 ± 0.02	0.79 ± 0.06	0.79 ± 0.02	0.533
Eggshell color	16.96 ± 2.84	17.91 ± 4.18	17.92 ± 2.98	17.07 ± 2.45	0.419
Yolk color fan score	9.44 ± 0.88 ^ab^	9.28 ± 1.00 ^a^	9.92 ± 1.00 ^bc^	10.45 ± 1.03 ^c^	<0.001
Haugh units	93.17 ± 6.34 ^a^	87.00 ± 9.60 ^b^	91.64 ± 6.96 ^a^	92.73 ± 5.93 ^a^	0.002
L*	70.90 ± 3.17 ^ab^	72.42 ± 3.43 ^a^	69.72 ± 6.86 ^b^	69.63 ± 4.0 ^b^	0.042
a*	9.40 ± 1.32 ^a^	8.01 ± 1.66 ^b^	11.47 ± 2.54 ^c^	12.24 ± 2.42 ^c^	<0.001
b*	55.52 ± 5.78 ^a^	56.53 ± 7.20 ^ab^	59.65 ± 7.63 ^b^	57.72 ± 5.54 ^ab^	0.056

T1: control; T2: basal diet with 1% MT extract; T3: basal diet with 2.5% MT extract; T4: basal diet with 4% MT extract. Values are means ± SD. a,b,c: values in the same row with different superscripts differ significantly (*p* < 0.05).

**Table 4:** Effect of dietary treatments (T1, T2, T3, T4) on egg yolk MDA (MDA) content and total phenol content (TPC) (*n* = 12 per treatment).

Parameters	Treatments				*p*-Value
	T1	T2	T3	T4	
MDA (ng MDA/g)	19.50 ± 15.69 ^b^	7.55 ± 5.22 ^a^	17.20 ± 13.76 ^ab^	15.73 ± 13.92 ^ab^	0.029
TPC (μg GAE/mL)	152.06 ± 11.71	179.14 ± 53.74	179.14 ± 53.74	168.32 ± 43.86	0.718

T1: control; T2: basal diet with 1% MT extract; T3: basal diet with 2.5% MT extract; T4: basal diet with 4% MT extract. Values are means ± SD. Values in the same row with different superscripts differ significantly (*p* < 0.05).

**Table 5:** Effect of dietary treatments (T1, T2, T3, T4) on egg yolk fatty acid profile (expressed in % of total fatty acid percentage) (*n* = 8 per treatment).

Fatty Acids (%)	Treatments				*p*-Value
	T1	T2	T3	T4	
Myristic (14:0)	0.26 ± 0.176 ^a^	0.29 ± 0.410 ^b^	0.26 ± 0.271 ^ab^	0.29 ± 0.304 ^ab^	0.075
Myristoleic (14:1n5)	0.04 ± 0.010 ^a^	0.07 ± 0.019 ^c^	0.05 ± 0.004 ^ab^	0.06 ± 0.034 ^b^	<0.001
Palmitic (16:0)	23.67 ± 0.680 ^a^	25.21 ± 1.430 ^b^	24.29 ± 0.500 ^a^	24.19 ± 1.100 ^ab^	0.005
cis-7-Hexadecenoic (16:1n9)	0.81 ± 0.220 ^b^	0.63 ± 0.100 ^a^	0.82 ± 0.118 ^b^	0.73 ± 0.066 ^ab^	0.041
Palmitoleic (16:1n7)	2.07 ± 0.415 ^a^	2.80 ± 0.740 ^b^	1.97 ± 0.167 ^a^	2.14 ± 0.213 ^a^	0.003
Margaric (17:0)	0.28 ± 0.516 ^b^	0.22 ± 0.274 ^a^	0.26 ± 0.055 ^b^	0.25 ± 0.214 ^ab^	0.033
cis-10-Heptadecenoic (17:1n7)	0.12 ± 0.026	0.10 ± 0.017	0.11 ± 0.023	0.10 ± 0.120	0.467
Stearic (18:0)	9.10 ± 1.108	9.94 ± 1.122	9.27 ± 1.131	9.02 ± 0.480	0.265
Elaidic (18:1n9t)	0.13 ± 0.018 ^a^	0.21 ± 0.035 ^b^	0.14 ± 0.022 ^a^	0.13 ± 0.147 ^a^	<0.001
Oleic (18:1n9)	38.09 ± 1.638 ^a^	42.02 ± 3.930 ^b^	39.71 ± 5.025 ^ab^	36.77 ± 1.106 ^a^	0.024
cis-Vaccenic (18:1n7c)	1.67 ± 0.115 ^a^	1.96 ± 0.265 ^b^	1.73 ± 0.152 ^a^	1.67 ± 0.074 ^a^	0.005
Linelaidic (18:2n6t)	0.04 ± 0.011 ^b^	0.03 ± 0.010 ^a^	0.04 ± 0.013 ^ab^	0.04 ± 0.005 ^ab^	0.077
Linoleic (18:2n6c)	18.10 ± 1.426 ^b^	11.34 ± 2.866 ^a^	16.71 ± 5.173 ^b^	18.77 ± 1.294 ^b^	<0.001
Arachidic (20:0)	0.15 ± 0.023 ^a^	0.14 ± 0.032 ^a^	0.16 ± 0.032 ^ab^	0.18 ± 0.010 ^b^	0.014
γ-Linolenic (18:3n6)	0.02 ± 0.003 ^ab^	0.01 ± 0.003 ^a^	0.02 ± 0.005 ^bc^	0.02 ±0.004 ^c^	<0.001
α-Linolenic (18:3n3)	0.82 ± 0.170 ^b^	0.49 ± 0.248 ^a^	0.63 ± 0.299 ^a^	0.80 ± 0.127 ^b^	0.018
all-cis-6,9,12,15-Octadecatetraenoic (18:4n3)	0.03 ± 0.009 ^a^	0.04 ± 0.011 ^b^	0.02 ± 0.006 ^a^	0.02 ± 0.006 ^a^	0.009
Gondoic (20:1n9)	0.26 ± 0.225	0.26 ± 0.047	0.27 ± 0.049	0.24 ± 0.016	0.557
Heneicosanoic (21:0)	0.02 ± 0.040	0.02 ± 0.003	0.02 ± 0.003	0.01 ± 0.004	0.315
cis,cis-11,14-Eicosadienoic (20:2n6)	0.21 ± 0.050 ^b^	0.11 ± 0.033 ^a^	0.20 ± 0.085 ^b^	0.20 ± 0.023 ^b^	0.004
Behenic (22:0)	0.05 ± 0.008	0.05 ± 0.008	0.04 ± 0.011	0.04 ± 0.004	0.118
all-cis-8,11,14-Eicosatrienoic (20:3n6)	0.14 ± 0.019	0.14 ± 0.027	0.15 ± 0.019	0.15 ± 0.008	0.829
all-cis-11,14,17-Eicosatrienoic (20:3n3)	0.02 ± 0.002	0.02 ± 0.005	0.02 ± 0.007	0.02 ± 0.008	0.470
Arachidonic (20:4n6)	2.10 ± 0.267	2.06 ± 0.149	2.14 ± 0.105	2.14 ± 0.087	0.723
Tricosanoic (23:0)	0.03 ± 0.008	0.03 ± 0.004	0.03 ± 0.004	0.03 ± 0.008	0.121
all-cis-5,8,11,14,17-Eicosapentaenoic (20:5n3)	0.02 ± 0.005	0.03 ± 0.009	0.02 ± 0.004	0.03 ± 0.008	0.222
all-cis-7,10,13,16-Docosatetraenoic (22:4n6)	0.23 ± 0.080	0.20 ± 0.048	0.21 ± 0.037	0.22 ± 0.034	0.699
all-cis-4,7,10,13,16-Docosapentaenoic (22:5n6)	0.25 ± 0.096	0.34 ± 0.140	0.27 ± 0.093	0.30 ± 0.071	0.313
all-cis-7,10,13,16,19-Docosapentaenoic (22:5n3)	0.14 ± 0.052	0.16 ± 0.083	0.13 ± 0.057	0.16 ± 0.050	0.808
all-cis-4,7,10,13,16,19-Docosahexaenoic (22:6n3)	1.13 ± 0.144	1.07 ± 0.247	1.02 ± 0.084	1.17 ± 0.148	0.326

T1: control; T2: basal diet with 1% MT extract; T3: basal diet with 2.5% MT extract; T4: basal diet with 4% MT extract. Values are means ± SD. Values in the same row with different superscripts differ significantly (*p* < 0.05).

**Table 6:** Effect of dietary treatments (T1, T2, T3, T4) on the macroscopical evaluation of the color, the size, the texture, and the sum of the aforementioned parameters, noted as Macro sum, on each treatment presented as number of counts and as percentages (%) within and among the treatments (*n* = 7 per treatment).

Parameter		Treatments				
**Color**		**T1**	**T2**	**T3**	**T4**	***p*-Value (X^2^Value)**
0	Count	0 ^a^	3 ^a^	0 ^a^	2 ^a^	0.013
	% within Group	0.00%	42.90%	0.00%	28.60%	
1	Count	3 ^a^	4 ^a,b^	7 ^b^	2 ^a^	
	% within Group	42.90%	57.10%	100.00%	28.60%	
2	Count	4 ^a^	0 ^b^	0 ^b^	3 ^a,b^	
	% within Group	57.10%	0.00%	0.00%	42.90%	
**Size**		**T1**	**T2**	**T3**	**T4**	***p*-Value (X^2^Value)**
0	Count	6	7	7	6	0.541
	% within Group	85.70%	100.00%	100.00%	85.70%	
1	Count	1	0	0	1	
	% within Group	14.30%	0.00%	0.00%	14.30%	
**Texture**		**T1**	**T2**	**T3**	**T4**	***p*-Value (X^2^Value)**
0	Count	3	5	2	3	0.309
	% within Group	42.90%	71.40%	28.60%	42.90%	
1	Count	3	2	5	2	
	% within Group	42.90%	28.60%	71.40%	28.60%	
2	Count	1	0	0	2	
	% within Group	14.30%	0.00%	0.00%	28.60%	
**Macro Sum**		**T1**	**T2**	**T3**	**T4**	***p*-Value (X^2^Value)**
0	Count	0	3	0	2	0.059
	% within Group	0.00%	42.90%	0.00%	28.60%	
1	Count	3	2	2	1	
	% within Group	42.90%	28.60%	28.60%	14.30%	
2	Count	0 ^x^	2 ^x,y^	5 ^y^	1 ^x^	
	% within Group	0.00%	28.60%	71.40%	14.30%	
3	Count	3	0	0	1	
	% within Group	42.90%	0.00%	0.00%	14.30%	
4	Count	0	0	0	1	
	% within Group	0.00%	0.00%	0.00%	14.30%	
5	Count	1	0	0	1	
	% within Group	14.30%	0.00%	0.00%	14.30%	

T1: control; T2: basal diet with 1% MT extract; T3: basal diet with 2.5% MT extract; T4: basal diet with 4% MT extract. Values are means ± SD. a,b: values in the same row with different superscripts differ significantly (*p* < 0.05). x,y: values in the same row with different superscripts tend to differ between them (0.05 < *p* < 0.1).

## Data Availability

The raw data supporting the conclusions of this article will be made available by the authors on request.

## References

[1] Windhorst H.-W. (2006). Changes in poultry production and trade worldwide. World’s Poult. Sci. J..

[2] Shini A., Shini S., Bryden W. (2019). Fatty liver haemorrhagic syndrome occurrence in laying hens: Impact of production system. Avian Pathol..

[3] Julian R.J. (2005). Production and growth related disorders and other metabolic diseases of poultry—A review. Vet. J..

[4] Leeson S. (2007). Metabolic challenges: Past, present, and future. J. Appl. Poult. Res..

[5] Trott K.A., Giannitti F., Rimoldi G., Hill A., Woods L., Barr B., Anderson M., Mete A. (2014). Fatty liver hemorrhagic syndrome in the backyard chicken: A retrospective histopathologic case series. Vet. Pathol..

[6] Rozenboim I., Mahato J., Cohen N.A., Tirosh O. (2016). Low protein and high-energy diet: A possible natural cause of fatty liver hemorrhagic syndrome in caged White Leghorn laying hens. Poult. Sci..

[7] Zaefarian F., Abdollahi M.R., Cowieson A., Ravindran V. (2019). Avian liver: The forgotten organ. Animals.

[8] Wu X.L., Zou X.Y., Zhang M., Hu H.Q., Wei X.L., Jin M.L., Cheng H.W., Jiang S. (2021). Osteocalcin prevents insulin resistance, hepatic inflammation, and activates autophagy associated with high-fat diet-induced fatty liver hemorrhagic syndrome in aged laying hens. Poult. Sci..

[9] Abenavoli L., Aviello G., Capasso R., Milic N., Capasso F. (2011). Milk thistle for treatment of nonalcoholic fatty liver disease. Curr. Med. Chem..

[10] Wu J.-W., Lin L.-C., Tsai T.-H. (2009). Drug-drug interactions of silymarin on the perspective of pharmacokinetics. J. Ethnopharmacol..

[11] Abenavoli L., Milic N. (2017). Silymarin for liver disease. Liver Pathophysiology.

[12] Pradhan S.C., Girish C. (2006). Hepatoprotective herbal drug, silymarin from experimental pharmacology to clinical medicine. Indian J. Med. Res..

[13] Milic N., Milosević N., Suvajdzić L., Zarkov M., Abenavoli L. (2013). New therapeutic potentials of milk thistle (*Silybum marianum*). Nat. Prod. Commun..

[14] Abenavoli L., Milic N., Capasso F. (2012). Anti-oxidant therapy in non-alcoholic fatty liver disease: The role of silymarin. Endocrine.

[15] De La Puerta R., Martinez E., Bravo L., Ahumada M.C. (1996). Effect of silymarin on different acute inflammation models and on leukocyte migration. J. Pharm. Pharmacol..

[16] Bosisio E., Benelli C., Pirola O. (1992). Effect of the flavanolignans of *Silybum marianum* L. on lipid peroxidation in rat liver microsomes and freshly isolated hepatocytes. Pharmacol. Res..

[17] Bannwart C.F., Peraçoli J.C., Nakaira-Takahagi E., Peraçoli M.T. (2010). Inhibitory effect of silibinin on tumor necrosis factor-alpha and hydrogen peroxide production by human monocytes. Nat. Prod. Res..

[18] Abenavoli L., Capasso R., Milic N., Capasso F. (2010). Milk thistle in liver diseases: Past, present, future. Phytother. Res..

[19] Bousserouel S., Bour G., Kauntz H., Gossé F., Marescaux J., Raul F. (2012). Silibinin inhibits tumor growth in a murine orthotopic hepatocarcinoma model and activates the TRAIL apoptotic signaling pathway. Anticancer Res..

[20] Wolford J., Polin D. (1975). Effect of inositol, lecithin, vitamins (B12 with choline and E), and iodinated casein on induced fatty liver-hemorrhagic syndrome in laying chickens. Poult. Sci..

[21] March B. (1981). Choline supplementation of layer diets containing soybean meal or rapeseed meal as protein supplement. Poult. Sci..

[22] Ruiz N., Miles R.D., Wilson H.R., Harms R.H. (1983). Choline supplementation in the diets of aged White Leghorn hens grouped according to body weight. Poult. Sci..

[23] Nesheim M.C., Norvell M.J., Ceballos E., Leach R.M. (1971). The effect of choline supplementation of diets for growing pullets and laying hens. Poult. Sci..

[24] Tsiagbe V., Kang C., Sunde M. (1982). The effect of choline supplementation in growing pullet and laying hen diets. Poult. Sci..

[25] Fischer L.M., daCosta K.A., Kwock L., Stewart P.W., Lu T.S., Stabler S.P., Allen R.H., Zeisel S.H. (2007). Sex and menopausal status influence human dietary requirements for the nutrient choline. Am. J. Clin. Nutr..

[26] Buchman A.L., Dubin M.D., Moukarzel A.A., Jenden D.J., Roch M., Rice K.M., Gornbein J., Ament M.E. (1995). Choline deficiency: A cause of hepatic steatosis during parenteral nutrition that can be reversed with intravenous choline supplementation. Hepatology.

[27] Kavan B.P., Khosravinia H., Karimirad R., Tavakolinasab F. (2023). Effects of dietary supplementation of milk thistle and nettle essential oils on performance, egg quality, and hematological parameters in layer hens. Poultry Sci. J..

[28] Karamali A., Parizadian Kavan B., Khaldari M., Masouri B. (2020). Effects of vitamin E and milk thistle powder (*Silybum marianum*) on liver health, serum parameters, and production indices in laying hens. Vet. Res. Biol. Prod..

[29] Quarantelli A., Romanelli S., Basini G., Righi F. (2009). The effects of Silymarin on ovarian activity and productivity of laying hens. Ital. J. Anim. Sci..

[30] Faryadi S., Sheikhahmadi A., Farhadi A., Nourbakhsh H. (2021). Effects of silymarin and nano-silymarin on performance, egg quality, nutrient digestibility, and intestinal morphology of laying hens during storage. Ital. J. Anim. Sci..

[31] (1999). Council Directive 99/74/EC of 19 July 1999 Laying down minimum standards for the protection of laying hens. Off. J. Eur. Communities.

[32] Shang H., Zhang H., Guo Y., Wu H., Zhang N. (2020). Effects of inulin supplementation in laying hens’ diet on the antioxidant capacity of refrigerated stored eggs. Int. J. Biol. Macromol..

[33] Folch J., Lees M., Sloane Stanley G.H. (1957). A simple method for the isolation and purification of total lipides from animal tissues. J. Biol. Chem..

[34] Aziza A.E., Awadin W., Cherian G. (2019). Impact of Choline Supplementation on Hepatic Histopathology, Phospholipid Content, and Tocopherol Status in Layer Hens Fed Flaxseed. J. Appl. Poult. Res..

[35] Corbin K.D., Zeisel S.H. (2012). Choline metabolism provides novel insights into nonalcoholic fatty liver disease and its progression. Curr. Opin. Gastroenterol..

[36] Lin C.W., Huang T.W., Peng Y.J., Lin Y.Y., Mersmann H.J., Ding S.T. (2021). A novel chicken model of fatty liver disease induced by high cholesterol and low choline diets. Poult. Sci..

[37] Dong X.F., Zhai Q.H., Tong J.M. (2019). Dietary choline supplementation regulated lipid profiles of egg yolk, blood, and liver and improved hepatic redox status in laying hens. Poult. Sci..

[38] Salamone F., Galvano F., Marino Gammazza A., Paternostro C., Tibullo D., Bucchieri F., Mangiameli A., Parola M., Bugianesi E., Li Volti G. (2012). Silibinin improves hepatic and myocardial injury in mice with nonalcoholic steatohepatitis. Dig. Liver Dis..

[39] Zhang W., Wang D., Hao E., Shi L., Chen H., Zhang W., Chen Y. (2024). Positive effects and mechanism of mulberry leaf extract on alleviating fatty liver hemorrhagic syndrome in laying hens. Poult. Sci..

[40] Hashemi Jabali N., Mahdavi A.H., Ansari Mahyari S., Sedghi M., Akbari Moghaddam Kakhki R. (2018). Effects of milk thistle meal on performance, ileal bacterial enumeration, jejunal morphology, and blood lipid peroxidation in laying hens fed diets with different levels of metabolizable energy. J. Anim. Physiol. Anim. Nutr..

[41] Šťastník O., Mrkvicová E., Pavlata L., Roztočilová A., Umlášková B., Anzenbacherová E. (2019). Performance, biochemical profile, and antioxidant activity of hens supplemented with addition of milk thistle (*Silybum marianum*) seed cakes in diet. Acta Univ. Agric. Silvic. Mendel. Brun..

[42] Omana D.A., Wang J., Wu J. (2010). Ovomucin—A glycoprotein with promising potential. Trends Food Sci. Technol..

[43] Kovacs-Nolan J., Phillips M., Mine Y. (2005). Advances in the value of eggs and egg components for human health. J. Agric. Food Chem..

[44] Liu X., Liu W., Deng Y., He C., Xiao B., Guo S., Zhou X., Tang S., Qu X. (2020). Use of encapsulated *Bacillus subtilis* and essential oils to improve antioxidant and immune status of blood and production and hatching performance of laying hens. Ital. J. Anim. Sci..

[45] Gao X., Xiao Z.H., Liu M., Zhang N.Y., Khalil M.M., Gu C.Q., Qi D.S., Sun L.H. (2018). Dietary silymarin supplementation alleviates zearalenone-induced hepatotoxicity and reproductive toxicity in rats. J. Nutr..

[46] Rama Rao S., Prashanth K., Paul S.S., Raju M.V.L.N., Nagalakshmi D., Prakash B. (2020). Evaluation of feeding value of combination of alternate protein sources in White Leghorn layers. Br. Poult. Sci..

[47] Surai P.F. (2015). Silymarin as a natural antioxidant: An overview of the current evidence and perspectives. Antioxidants.

[48] Obianwuna U.E., Oleforuh-Okoleh V.U., Wang J., Zhang H.J., Qi G.H., Qiu K., Wu S.G. (2022). Potential implications of natural antioxidants of plant origin on oxidative stability of chicken albumen during storage: A review. Antioxidants.

[49] Surai P.F., Speake B.K., Sparks N.H.C. (2001). Carotenoids in avian nutrition and embryonic development. 1. Absorption, availability and levels in plasma and egg yolk. J. Poult. Sci..

[50] Dansou D.M., Zhang H., Yu Y., Wang H., Tang C., Zhao Q., Qin Y., Zhang J. (2023). Carotenoid enrichment in eggs: From biochemistry perspective. Anim. Nutr..

[51] Taleb A., Ahmad K.A., Ihsan A.U., Qu J., Lin N., Hezam K., Koju N., Hui L., Qilong D. (2018). Antioxidant effects and mechanism of silymarin in oxidative stress-induced cardiovascular diseases. Biomed. Pharmacother..

[52] Islam K.M.S., Khalil M., Manner K., Raila J., Rawel H., Zentek J., Schweigert F.J. (2017). Lutein specific relationships among some spectrophotometric and colorimetric parameters of chicken egg yolk. J. Poult. Sci..

[53] Failla M.L., Chitchumronchokchai C., Ferruzzi M.G., Goltz S.R., Campbell W.W. (2014). Unsaturated fatty acids promote bioaccessibility and basolateral secretion of carotenoids and α-tocopherol by Caco-2 cells. Food Funct..

[54] Papadopoulos G.A., Chalvatzi S., Kopecký J., Arsenos G., Fortomaris P.D. (2019). Effects of dietary fat source on lutein, zeaxanthin, and total carotenoids content of the egg yolk in laying hens during the early laying period. Br. Poult. Sci..

[55] Cherian G., Gonzalez D., Ryu K., Goeger M. (2007). Long-term feeding of conjugated linoleic acid and fish oil to laying hens: Effects on hepatic histopathology, egg quality, and lipid components. J. Appl. Poult. Res..

[56] Cherian G. (2008). Egg quality and yolk polyunsaturated fatty acid status in relation to broiler breeder hen age and dietary n-3 oils. Poult. Sci..

[57] Grotto D., Maria L.S., Valentini J., Paniz C., Schmitt G., Garcia S.C., Pomblum V.J., Rocha J.B.T., Farina M. (2009). Importance of the lipid peroxidation biomarkers and methodological aspects for malondialdehyde quantification. Quim. Nova.

[58] Gholamalian R., Mahdavi A.H., Riasi A. (2021). Hepatic fatty acids profile, oxidative stability and egg quality traits ameliorated by supplementation of alternative lipid sources and milk thistle meal. J. Anim. Physiol. Anim. Nutr..

[59] Lemahieu C., Bruneel C., Ryckebosch E., Muylaert K., Buyse J., Foubert I. (2015). Impact of different omega-3 polyunsaturated fatty acid (n-3 PUFA) sources (flaxseed, Isochrysis galbana, fish oil, and DHA Gold) on n-3 LC-PUFA enrichment (efficiency) in the egg yolk. J. Funct. Foods.

